# Design, characterizations, and antimicrobial activity of sustainable home furnishing-based waste fabric treated using biobased nanocomposite

**DOI:** 10.1186/s40643-024-00787-z

**Published:** 2024-07-25

**Authors:** Eman M. Swielam, Zeinab M. Hussien, Mohamed S. Hasanin

**Affiliations:** 1 Clothing and Knitting Industrial Research Department, Textile Research and Technology Institute, National Research Centre, Dokki, Cairo 12622 Egypt; 2 Ready Made Garments Division, Industrial Arts Department, Faculty of Education, Helwan University, Cairo, Egypt; 3 Cellulose and Paper Department. Chemical Industries Institute, National Research Centre, Dokki, Cairo 12622 Egypt

**Keywords:** Textile sustainability, Recycling, Home products, Chitosan, Antimicrobial activity

## Abstract

Clothing and textile industries are major contributors to environmental pollution including textile manufacturing through garment production, spinning, weaving, and dyeing. In this context, the sustainability textile industry is a big challenge and contributes to serving a large segment of society. Also, textile wastes could be used as a raw material for added-value products. Herein, in this study, recycling of residues fabric was treated with antimicrobial nanocomposite to reach the best use of exhausts and obtain multifunction products of aesthetic via the technical design of the waste raw materials. Besides, solving the unemployment problem by opening fields for small industry projects capable of producing high-value textile artifacts, especially when treated against microbes, can be applied to home furnishings. The waste fabric was treated via green synthesis nanocomposite based on chitosan and in situ prepared ZnONPs and cross-linked with tannic acid. The prepared nanocomposite was characterized using physicochemical analysis including attenuated total reflectance Fourier transform infrared spectroscopy (ATR-FTIR) and X-ray diffraction (XRD). Additionally, the nanocomposite and treated fabric topographical behavior were studied using scanning electron microscopy (SEM) attachment with energy dispersive X-ray analysis (EDX), and images were processed to evaluate the roughness structure. Additionally, high-resolution transmission electron microscopy (HR-TEM) and dynamic light scattering (DLS) were performed to ensure the size and stability of the nanocomposite. The obtained results affirmed the green synthesis of nanocomposite with a size around 130 nm, as well as the doped ZnONPs average size of 26 nm and treated waste fabric, performed a promising attraction between nanocomposite and fabric fibers. Moreover, the antimicrobial study observed excellent activity of nanocomposite against bacteria and unicellular fungi as well.

**Supplementary Information:**

The online version contains supplementary material available at 10.1186/s40643-024-00787-z.

## Introduction

The textile industry is one of the most polluting industries in the world (Costa et al. 2020). Worthwhile, from raw materials extraction to the final disposal of garments or home furnishings, every stage of the textile life cycle hurts the environment to some degree (de Villiers et al. 2014). Instead, many innovative ideas, thoughts, technologies, actions, solutions, and policies are available to minimize the negative impact of the industry, the industry is nowhere near achieving a satisfactory environmental profile (Legrand et al. 2022; Patwary 2020). The textile industry is an important major sector for economic development in countries all over the world (Islam et al. 2013). Indeed, it has a complicated structure with complex production chains and many different sub-sectors (Bullon et al. 2017). The textile industry is also famous for its intensive resource usage in complex production processes and high quantity of chemical consumption, especially in dyeing and finishing processes (Islam et al. 2023; Saxena et al. 2017; Zhang et al. 2023). Specific consumption can vary depending on fiber type and applied technologies in textile production processes (Fathy 2016).

Home furnishings are one of the most widely used textile materials in many areas, and their application in furniture environments can produce various results (Xiong et al. 2020). Besides, they can be made light and soft, or colorful (Yamini Jhanji 2022). Different individuals prefer different atmospheres, and thus require different ways of applying textiles, in addition to the importance of being antimicrobial (Gulati et al. 2022; Karim et al. 2020). Textiles play a growing role in home products toward these considerations (Ahmad et al. 2020). They impart a more flexible aspect to even the most rigid home furniture materials especially when recycled exhausted to reduce the waste to achieve sustainability, Sustainable design with waste fabric takes us full circle back to the goal of avoiding pollution (Javaid et al. 2021; Vezzoli and Manzini 2008).

Indeed, the recycled fabric waste of home furnishings manufacturing can be divided according to the source: waste from the spinning, waste from the weaving, dyeing, and finishing waste stage, and manufacturing waste stage are considered zero-cost byproducts as well (Akter et al. 2022; Kamble and Behera 2021; Lu et al. 2023). On the other side, the treatment of waste textiles could be an effective strategy for the valorization of these waste products. Many promising materials are used to gain waste textiles a unique feature that includes; nanomaterials, polymers and dye (Patti et al. 2020). Unfortunately, these materials usually produce eco-friendly products that contain nonbiodegradable resides as well as hazardous materials that affect the global environment (Olaiya et al. 2022). In this manner, the biopolymers and green synthesis nanomaterials are strongly fit materials for sustainable waste textile treatments (Elsayed et al. 2021; Hasanin 2022). Chitosan is one of the biopolymers that is characterized by amin groups that present antimicrobial activity that also, could be used as a capping and stabilizing agent for nanoparticle production (Al Kiey et al. 2022; Hasanin et al. 2021, 2022, 2023a).

On the other hand, zinc oxide nanoparticles (ZnONPs) are a biocompatible nano metal oxide that is characterized by a high safety profile as well as can be synthesis via a green method in which the biopolymer such as chitosan could be used as a capping and stabilizing agent to prepare (Elsayed et al. 2022; Abdelhameed et al. 2022). Besides, some materials such as natural organic acids are used to assist the green biosynthesis of nanometals such as tannic acid (Mallakpour et al. 2023), salicylic acid (Pant et al. 2018), and critic acid (Pooresmaeil et al. 2022). Tannic acid is a type of polyphenol that functions as a mild acid (Baldwin and Booth 2022). On the twigs of some oak trees, tannic acid is present in the nutgalls that insects produce (Quercus infectoria and other Quercus species). It is taken out and utilized medicinally (Yan et al. 2020). Therefore, in this presented work waste textile was treated with nanocomposite chitosan and ZnONPs that were prepared via a green method using chitosan itself as a capping and stabilizing agent as well as the tannic acid was used as an assisted capping and reducing agent. The waste textile was designed and treated with a nanocomposite as well and the nanocomposite was characterized via a physicochemical analysis and the treated textile was characterized via a topographical analysis and mechanical properties were estimated.

## Materials and methods

### Materials

Waste blended fabric from cotton and polyester with a percentage of 65% and 35%, respectively for embroidered applique, cotton fabric with a percentage of 95% for basic cover material also for pillows, cushions and satin thread for embroidery. Chitosan (Ch) used in this study was purchased from Sigma Aldrich (St. Louis, and molecular weight 650,000, viscosity, 275.9 cps, and degree of deacetylation, 85.5%. Zinc acetate was purchased from Lob Chem, India. All chemicals, reagents, and media used were in analytical grade without any purification required before use. Tannic acid was purchased from Sigma Aldrich, Germany.

### Preparation of nanocomposite and fabric treatment

A solution of 1% w/v chitosan dissolved in 1% w/v acetic acid (100mL) was added to a 100 mL solution containing zinc acetate (1 mol) and left under stirring at 1500 rpm for 1 h at 70°C. Afterward, 12 mg of tannic acid was added and stirred at the same conditions for 1 h. Finally, the waste textile was impressed in the above-prepared solution and ultrasonicated in the ultrasonic water bath for 1 h at 70°C. The produced treated textile was dried in the oven at 70°C overnight.

### Characterizations

ATR-FTIR (Spectrum Two IR Spectrometer - PerkinElmer, Inc., Shelton, USA). All spectra were obtained by 32 scans and 4 cm^− 1^ resolution in wavenumbers ranging from 4000 to 400 cm^− 1^. The XRD crystallographic patterns were investigated using a Diano X-ray diffractometer (Philips) provided with a CuKα radiation source (λ = 0.15418 nm), energized at 45 kV, as well as a generator (PW 1930) and a goniometer (PW 1820). The surface morphology was investigated by SEM Model Tescan Vega 3 SBU attached to an EDX Unit for EDX with an accelerating voltage of 20 kV. The surface roughness was investigated by the Gwyddion software Version (2.32) to transform the (2D) image into the (3D) image. Nnaostrcture was studied using high-resolution transmission electron microscopy (HR-TEM) (JEM-1230, JEOL, Tokyo, Japan and dynamic light scattering (DLS) was carried out for the nanocomposite sample using the instrument (Santa Barbara, CA, USA) that was utilized to ascertain the average size in conditions; 23 °C, with the incident light being the 632.8 nm line of a HeNe laser at an angle of 13.9°.

### Antimicrobial activity

Antimicrobial activity of nanocomposite as well as treated sample and chitosan as control were carried out using the turbidimetric method described in the previous works (Al Kiey et al. 2022; Elsayed et al. 2021) against three selected pathogenic microorganisms, including Gram-negative bacterial strains, namely; *Escherichia coli* ATCC25922, Gram-positive bacteria strain *Staphylococcus aureus* ATCC 25,923, and unicellular fungal strain, namely; *Candida albicans ATCC90028*. 100 mg of each tested sample was used as a specimen. The selected microorganisms were incubated in a nutrient broth medium for 24 h at 37 °C for bacteria with a concentration of about 1.5 × 10^8^ CFU/mL. The fungal strain was grown on potato dextrose broth medium and incubated at 30 °C for 3–5 days. In addition, the antimicrobial activity was repeated for washed fabric after 10 cycles with the same conditions as non-washed treated fabric.

### Cytotoxicity assay

The cytotoxic activity test was carried out using a normal cell line, BJ1 (normal skin fibroblast), and Vero fibroblast cells via MTT protocol (Riss et al. 2016) for nanocomposite sample. Cell viability was calculated as the number of cells and the percentage of viable cells was determined using Eq. (1):

1 $$\:\text{V}\text{i}\text{a}\text{b}\text{i}\text{l}\text{i}\text{t}\text{y}\:\text{\%}=\:\frac{\text{T}\text{e}\text{s}\text{t}\:\text{O}\text{D}}{\text{C}\text{o}\text{n}\text{t}\text{r}\text{o}\text{l}\:\text{O}\text{D}}\:\text{X}\:100\:\:\:\:\:\:\:\:\:\:\:\:\:\:\:\:\:\:\:\:\:\:\:\:\:\:\:\:\:\:\:\:\:\:\:\:\:\:\:\:\:\:\:\:\:\:\:\:\:\:\:\:\:\:\:\:\:\:\:\:$$

### Design of home furnishing

Design made suitable for sustainability based on the collected materials and its mechanical properties for cover bed and cushions with Adobe Illustrator design program version: CC.2022.v26.0.0.730.x64. Then an automatic embroidery method to attach selected waste materials on basic cotton covers and cushions with an applique technique to add aesthetic value besides the functional value.

### Mechanical properties

Collected different materials from waste fabric and made the mechanical properties tests to study the characterization for them such as; (Tensile strength, strain, bending rigidity, thickness, washing fastness and rubbing) to select the materials compatible with each other.

### Mechanical properties

Mechanical properties including; Tensile strength measured according to (ASTM - D 3822) (Instron) and bending rigidity of fabrics measured according to (ASTM - D 1388-96) Shirey stiffness tester,

### Physical measurements

Thickness in (mm) was measured according to (ASTM-D1777). Weight in (g/m²) according to (ASTM-D3776). Washing fastness: colorfastness to washing the colorfastness to washing, Light, rubbing, and perspiration measurements were determined according to the standard methods AATCC test method (AATCC Technical Manual, Method 36, (1972), 68, 23, (1993)) using Launder Ometer.

## Results and discussion

### Preparation of nanocomposite and fabric treatment

The preparation of nanocomposite was carried out via a green method in which the ZnONPs synthesis in situ via chitosan as a capping and stabilizing agent. In this context, the treatment of waste fabric was carried out via ultrasonication method to reduce the time and cost. Herein, the loading of nanocomposite into the fabric and cross-linked using tannic acid reflect a strong fixation on the fabric fibers.

### Characterizations

Characterizations of nanocomposite were included in the comparative study between chitosan and nanocomposite including physicochemical analysis (ATR-FTIR and XRD) to understand the changes during the green synthesis of nanocomposite. In addition, the attaching between nanocomposite and fabric was studied via SEM.

**Fig. 1:**
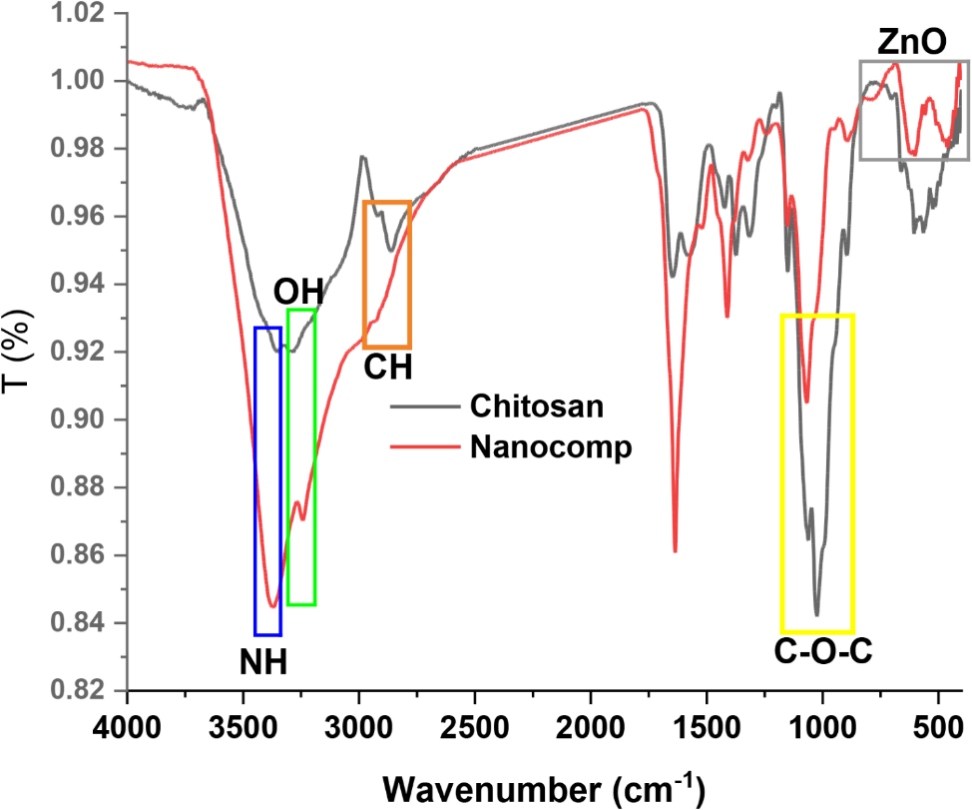
FTIR spectra of pure chitosan and prepared nanocomposite

AT-FTIR spectra of chitosan and nanocomposite were illustrated in Fig. 1. The Chitosan spectrum presented significant information about the formulation of the nanocomposite. The hydroxyl group position presented an extension in band intensity in the nanocomposite spectrum in comparison with chitosan and the hydroxyl band was shifted to low frequency and the NH band was shifted to high frequency with an increase in intensity as well. In addition, the CH stretching vibration band was reduced as a small band at a higher frequency in comparison with the chitosan spectrum. Moreover, the N-H bending band was merged in the nanocomposite in comparison with the chitosan spectrum. Moreover, the carbohydrate band in chitosan was recorded at 1011 cm^− 1^ and in nanocomposite at 1063 cm^− 1^ with low intensity (Hasanin et al. 2023b). These findings could be due to the effect of a combination of ZnO and chitosan. On the other hand, the ZnO bands were assigned at 760, 600 and 460 cm^− 1^ as obvious bands (Handore et al. 2014). In sum, these observations affirmed that the nanocomposite was prepared.

**Fig. 2:**
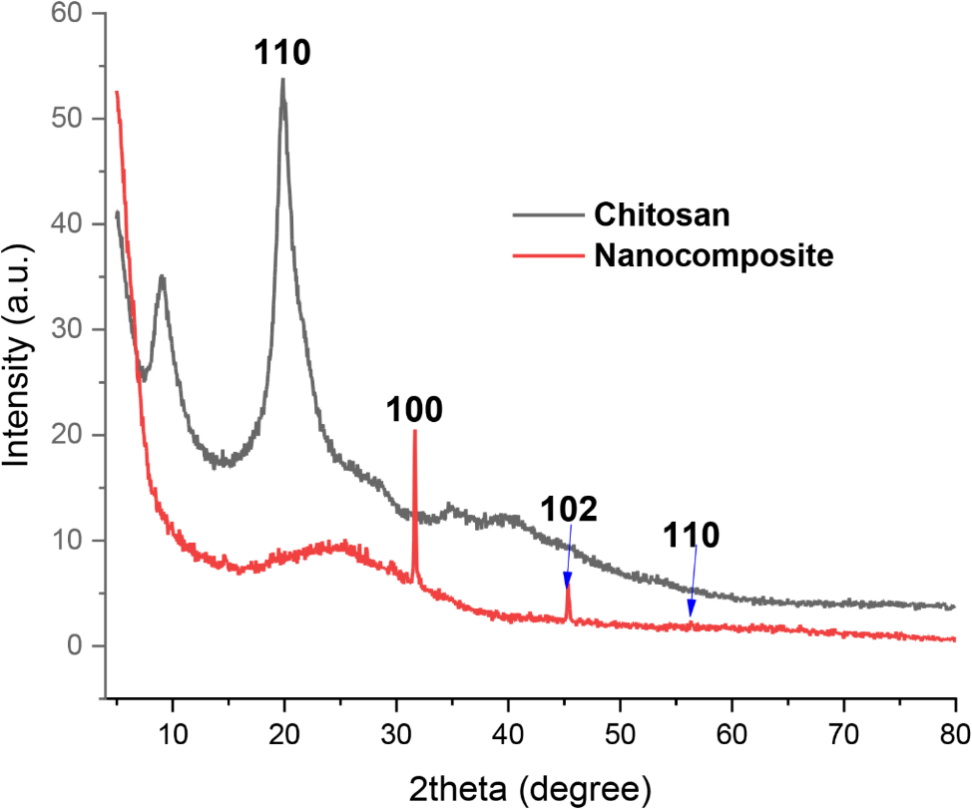
XRD patterns of pure chitosan and nanocomposite

The XRD patterns of chitosan and nanocomposite are presented in Fig. 2. The chitosan pattern was illustrated in two humps at 2Ɵ 10° and 20° corresponding to the Miller indices (110) were characteristic of pure chitosan (Hajjaji et al. 2022; Medany et al. 2024). Indeed, these humps were deleted after the preparation of nanocomposite this could be due to trapped ZnONPs into the chain skeleton of chitosan as well as the attaching of ZnONPs with function groups of chitosan. Moreover, the ZnONPs were presented as an obvious peak at 2Ɵ 31.6° and 45.4° corresponding to the Miller indices (100) and (102), respectively (Medany et al. 2024). However, that peak at 56.3° (110) was assigned as a small intensity this could be related to the trapping of ZnONPs into the chitosan chain (Hasanin et al. 2023a; Ishwarya et al. 2018). Additionally, these peaks were related to ZnO JCPDS card no 800 075 (Hitkari et al. 2017; Savaloni and Savari 2018).

**Fig. 3:**
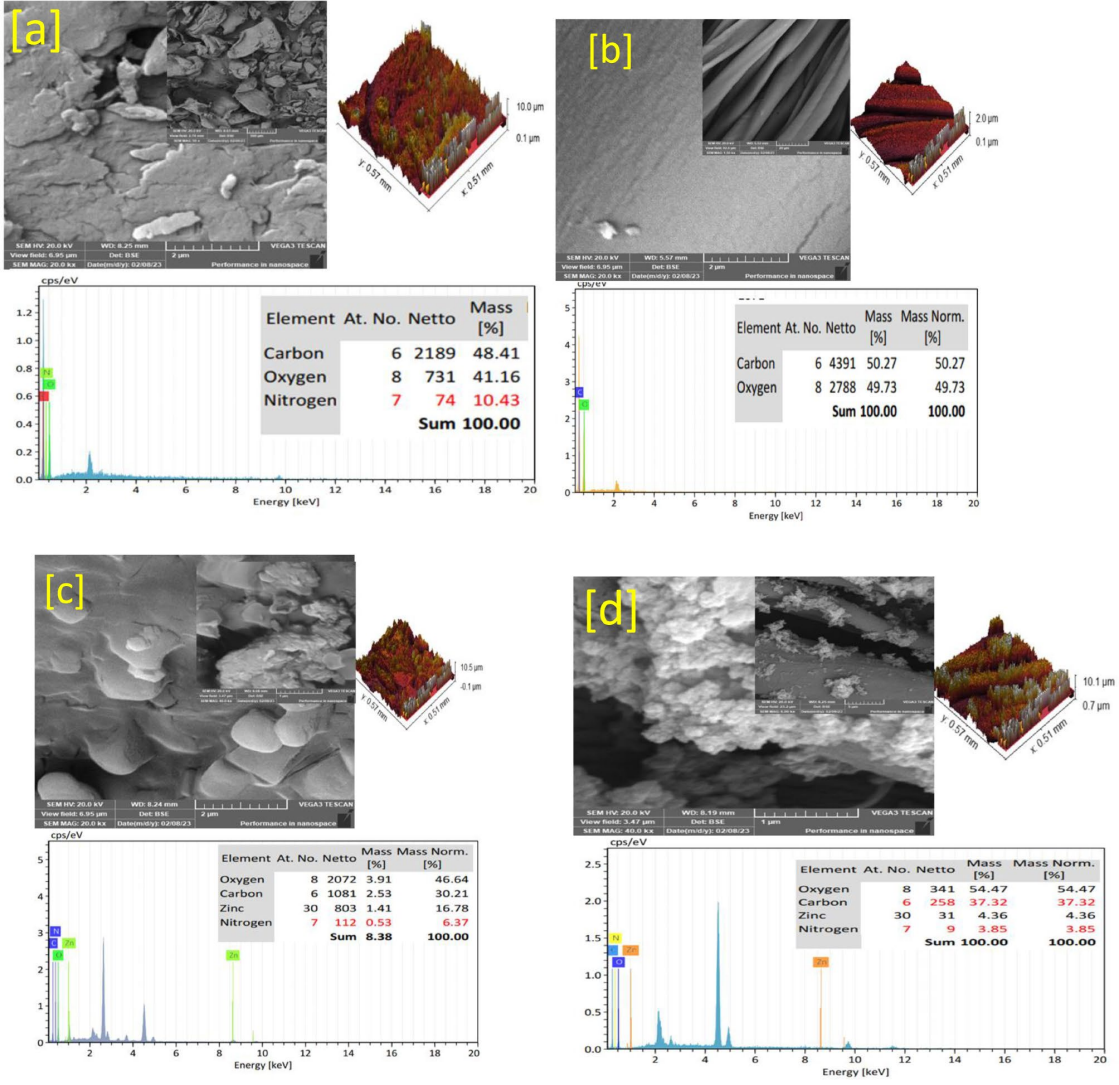
SEM, EDX and roughness of chitosan (**a**), fabric (**b**), nanocomposite (**c**) and treated fabric (**d**), respectively. Figure 4 illustrates the high-resolution TEM images as heterogenous

**Fig. 4:**
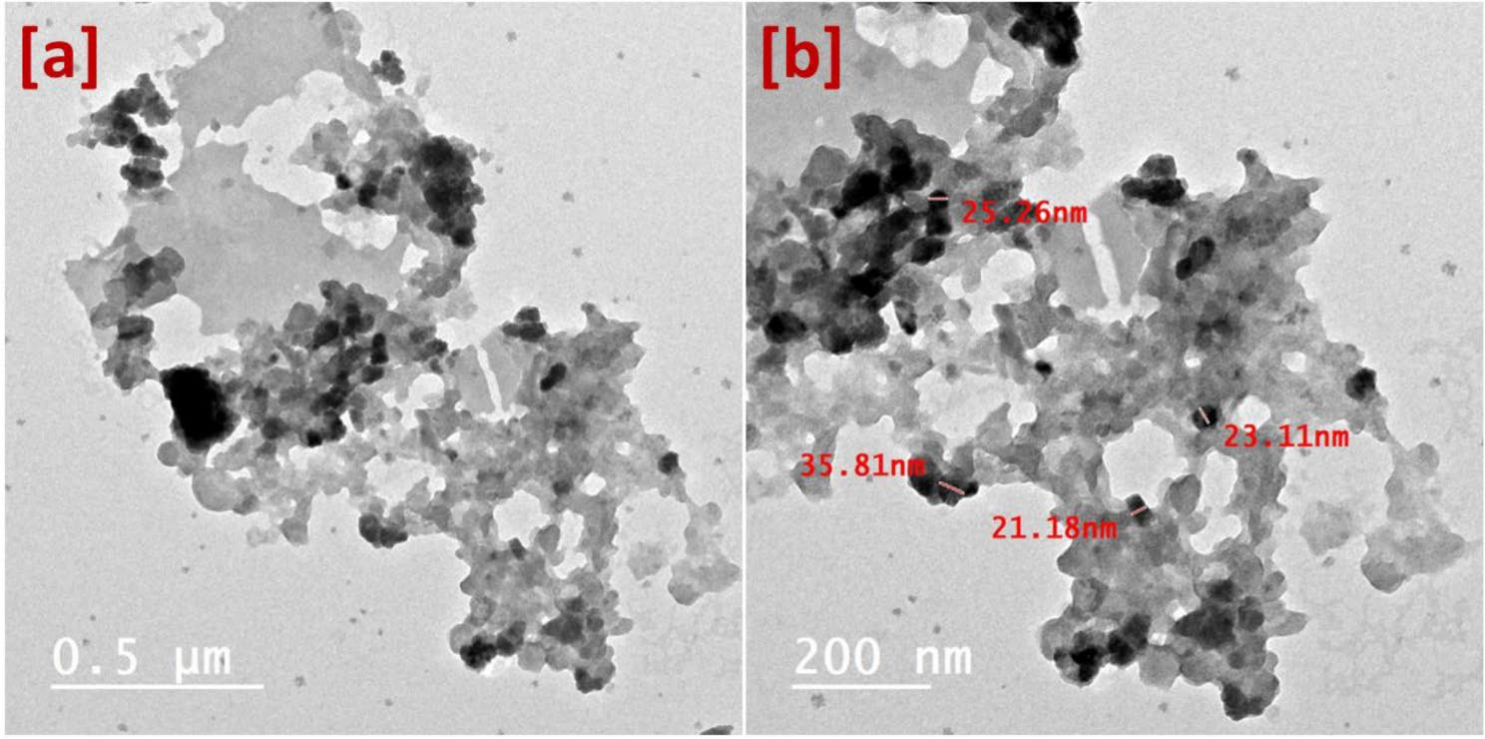
TEM images of nanocomposite: low magnification high-resolution TEM (**a**) and high magnification high-resolution TEM (**b**)

The topographical study presented the pure chitosan, pure fabric, nanocomposite and treated fabric in Fig. 3. Pure chitosan (Fig. 3a) presented a layer-like image with a cracked rough surface that supported the roughness process image that appears as a rough surface with many cracks and this is in good agreement with the pure chitosan SEM images in the literature (Sweah et al. 2020). The EDX chart presented carbon, oxygen, and nitrogen atoms. Pure fabric images (Fig. 3b) were observed as clusters of fibers-like shapes with smooth surfaces and the EDX chart presented carbon and oxygen atoms. The roughness image illustrated a smooth surface and supported the original SEM images. Moreover, the nanocomposite images (Fig. 3c) observed a unique surface morphology that did not look like chitosan with a cluster of metallic shine that referred to ZnONPs. In addition, the EDX chart of nanocomposite presented carbon, oxygen, nitrogen and zinc atoms as well as the roughness image affirmed the rough nature of the nanocomposite surface that could help the attaching with fabric fibers. Finally, the treated fabric SEM images were presented in Fig. 3d and observed the fibers loaded with nanocomposite and the surface roughness was affected the EDX chart also presented the presence of carbon, oxygen, nitrogen and zinc atoms. The topographical study emphasized the biosynthesis of ZnONPs and nanocomposite as well as observed the attachment of nanocomposite above the fabric fibers.

shapes of nanoparticles dispersed in the polymer matrix at low magnification TEM image (Fig. 4a). Moreover, the high magnification TEM image (Fig. 4b) observed ZnONPs as dark black nanoparticles with a size of about 26 nm. In this context, the nanocomposite was presented in a nanostructure as a matrix involving nanoparticles (ZnONPs).

DLS measurements of nanocomposite was including average particles size distribution, poly dispersing index (PDI), and average zeta potintial. The average particle size distribution was recorded at 137 nm with PDI 0.19 which reflected a nano range of nanoparticles above 100 nm according to the molecule size of chitosan. Moreover, PDI vale was affirmed as a good homogeneous of the nanocomposite. Indeed, these results are in a nice agreement with the SEM images. However, the high-resolution TEM image has illustrated the nanocomposite with an average nanosize of about 26 nm. This difference in the size comes as a result of the matrix that is detected in DLS measurements. In this context, the average zeta potential was recorded at -37 mV with nice stability of the nanocomposite.

### Antimicrobial activity study

The antimicrobial activity of treated fabric that comparison with pure one was tabulated in Table 1. The pure fabric presented no antimicrobial activity against all tested microbial strains. However, chitosan shows moderate antimicrobial activity against tested microbial strains due to the weak mechanism of action efficiency of pure chitosan which binds with bacteria cell walls and disruption (Guarnieri et al. 2022; Yilmaz Atay 2019). In addition, the nanocomposite presented excellent antimicrobial activity in comparison with pure chitosan against tested microbial strains that recorded about 90% for all microbes. This activity could be due to the synergetic effect between chitosan and ZnONPs that kills the microbial cells via disruption of the cell membrane and binding with effective cellular proteins and DNA as well (Gudkov et al. 2021). Moreover, the treated fabric presented a nice activity against bacteria strains that could be described as a broad-spectrum activity that affects Gram-positive and negative bacterial strains. Otherwise, the unicellular fungi were affected and the growth was reduced by about 84%. Finally, these results affirmed the antimicrobial activity of treated fabric that affects the bacteria and unicellular fungi. Indeed, the antimicrobial activity was gained from the synergetic effect between the chitosan antimicrobial activity and ZnONPs antimicrobial activity where both enhance each other and present a nice antimicrobial activity (Goy et al. 2009; Lallo da Silva et al. 2019). On the other side, the antimicrobial of treated fabric after 10 washing cycles recorded a decrease in antimicrobial activity to less than half-fold this finding emphasizes the good attachment of nanocomposite with fabric fires and confirms the topographical study.

**Table 1:** The antimicrobial activity of pure chitosan, nanocomposite, and treated fabric after and before 10 washing

Microbes	Without washing			After 10 washing cycles		
Samples	*E. coli*	*S. aureus*	*C. albicans*	*E. coli*	*S. aureus*	*C. albicans*
Fabric (Blank)	0*	0	0	0	0	0
Chitosan	36 ± 2.5	39 ± 3.0	40 ± 3.3	NA**	NA	NA
Nanocomposite	94 ± 3	92 ± 4.5	96 ± 3.7	NA	NA	NA
Treated fabric	86 ± 3.2	83 ± 2.8	84 ± 2.3	58 ± 4.2	59 ± 5.3	52 ± 4.5

*growth inhibition activity %

** not applicable for this sample

### Cytotoxicity assay

**Fig. 5:**
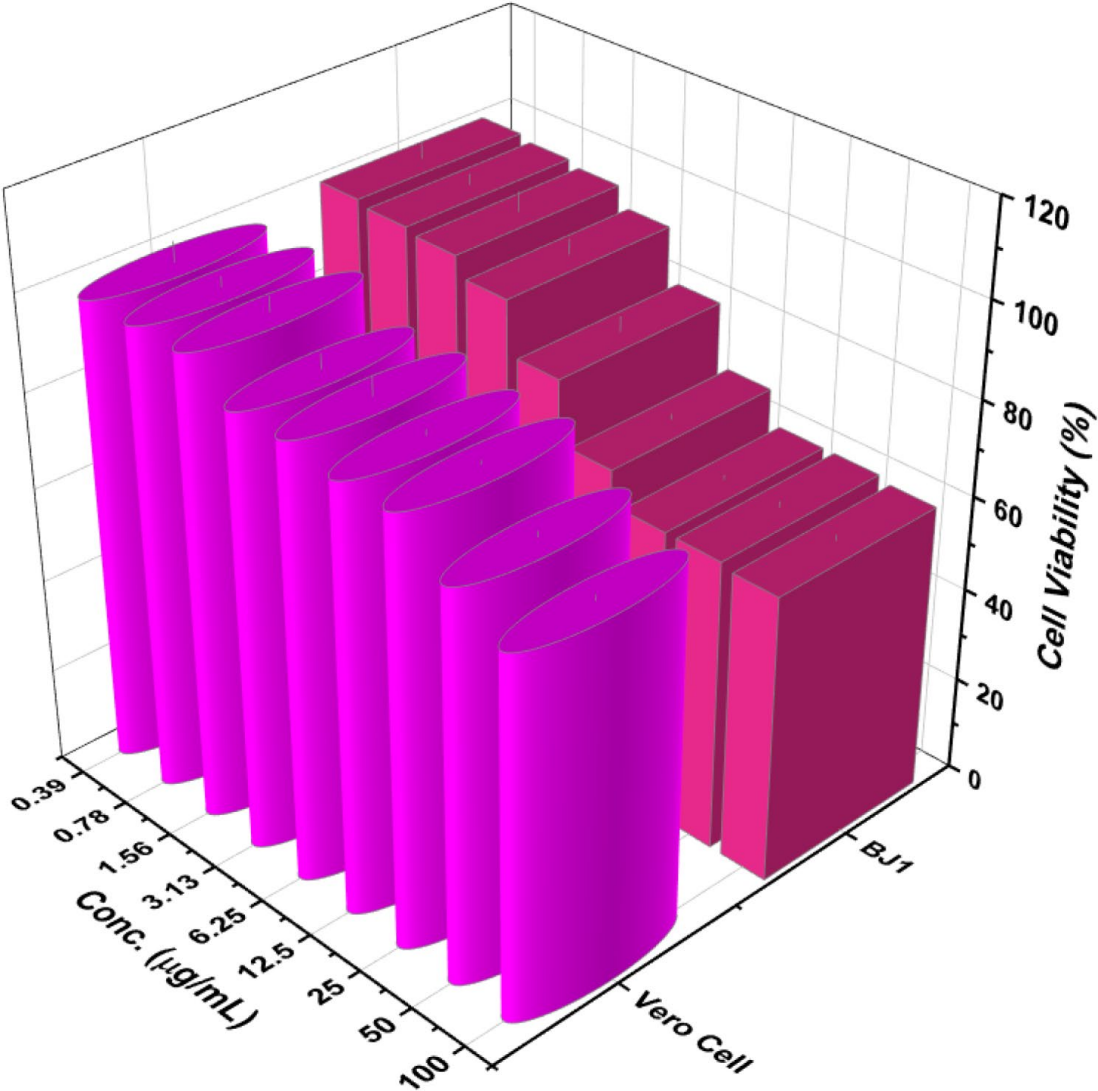
Cytotoxicity assay for formulated nanocomposite

Figure 5 illustrates the cytotoxicity of the nanocomposite against two normal cell lines. Indeed, the IC_50_ of nanocomposite toward two cell lines was recorded as lower than 100 µg/mL. These values were localized at the high safety area and these results were going in a direct direction with the biomolecules that were used in the formulation of nanocomposite. Chitosan (Frigaard et al. 2022) and tannic acid (Wu et al. 2022)were recorded with low cytotoxicity as well as the ZnONPs are considered dermo-compatible nanoparticles (Azizi et al. 2024).

### Mechanical measurements

The mechanical tests of the treated waste fabric were tabulated in Table 2 before treatment and in Table 3 after treatment. Some of the following mechanical properties tests were done to select the materials that are compatible with each other As shown in Table 2, the maximum load for samples in warp direction was between 51.80 and 61.52 kgf, and in weft direction between 49.19 and 55.26 kgf, maximum strain between 10.67 and 12.67% in warp direction and in weft direction was between 18.23 and 21.80%, Bending rigidity was 4.5 mm as the highest value and 4.1 mm as the lowest value in warp direction and was 4.7 mm as the highest value and 3.5 mm as the lowest value in weft direction, the thickness value between 0.27 mm and 0.31 mm and weight values between 88.9 and 90.98 g/m^2^.

**Table 2:** Tensile strength, strain, bending rigidity and thickness test before the treatment

No. fabric	Weave direction	Max. load (kgf)	Max. stress (kgf/mm^2^)	Max. strain (%)	Bending rigidity (mm)	Thickness (mm)	Weight (g/m^2^)
1	warp	51.80	5.180	10.67	4.2	0.31	90.8
	weft	49.19	4.919	21.33	3.5		
2	warp	61.52	6.152	12.67	4.5	0.27	88.9
	weft	55.26	5.526	18.67	4		
3	warp	52.91	5.291	10.98	4.3	0.30	90.3
	weft	50.02	5.002	21.75	3.9		
4	warp	51.99	5.199	10.77	4.5	0.28	90.02
	weft	50.15	5.015	21.80	4		
5	warp	52.62	5.262	11.03	4.1	0.30	90.6
	weft	50.98	5.098	19.65	3.6		
6	warp	55.35	5.535	11.95	4.2	0.29	90.00
	weft	53.56	5.356	18.23	3.6		
7	warp	56.23	5.623	11.98	4.3	0.31	90.98
	weft	54.15	5.415	18.89	4.7		

As shown in Table 3, the maximum load for samples in the warp direction was between 51.83 and 62.00 kgf and in the weft direction between 49.10 and 55.29 kgf, maximum strain between 10.66 and 12.67% in the warp direction and in weft direction was between 18.24 and 21.81%, Bending rigidity was 4.5 mm as the highest value and 4.2 mm as the lowest value in the warp direction and was 4.6 mm as the highest value and 3.6 mm as the lowest value in the weft direction, the thickness value between 0.28 mm and 0.32 mm and weight values between 89 and 91 g/m^2^. The results revealed as shown in Tables 2 and 3 that all samples had very good values for tensile strength and strain and nearly values in bending rigidity and thickness this indicates that the materials are compatible with each other before and after treatment not only for aesthetics value but in mechanical properties to ensure the quality of the final product (TURAN et al. 2023).

**Table 3:** Tensile strength, strain, bending rigidity and thickness test after the treatment

No. fabric	Weave direction	Max. load (kgf)	Max. stress (kgf/mm^2^)	Max. strain (%)	Bending rigidity (mm)	Thickness (mm)	Weight (g/m^2^)
1	warp	51.83	5.237	10.66	4.3	0.32	91.0
	weft	49.23	5.079	21.34	3.6		
2	warp	62.00	6.174	12.67	4.5	0.28	89.0
	weft	55.29	5.531	18.68	4.1		
3	warp	53.00	5.301	10.99	4.2	0.31	90.6
	weft	50.10	5.032	21.75	3.9		
4	warp	52.09	5.200	10.78	4.5	0.29	90.10
	weft	50.23	5.020	21.81	4.1		
5	warp	52.66	5.250	11.06	4.3	0.31	90.9
	weft	51.02	5.095	19.66	3.7		
6	warp	55.45	5.532	11.95	4.3	0.30	90.30
	weft	53.72	5.360	18.24	3.6		
7	warp	56.31	5.624	12.00	4.2	0.32	91.0
	weft	54.21	5.413	18.90	4.6		

As shown in Table 4 the results revealed that all samples had excellent fastness against washing. Samples no. 1, 4, 5, and 7 demonstrated excellent fastness and rubbing, fastness to rubbing values ranged from excellent to very good in samples no. 2, 3 and 6. Previously, we found that the results of all tests indicated that selected materials are compatible with each other in terms of mechanical properties, and All previous tests before and after treatment showed it has no significant effect on mechanical properties for all materials (Sadeghi-Kiakhani et al. 2018).

**Table 4:** Washing fastness and rubbing test before & after treatment

	Before					After				
No. fabric	Washing fastness			Rubbing		Washing fastness			Rubbing	
	St.		Alt.	Dry	Wet	St.		Alt.	Dry	Wet
	cotton	wool				cotton	wool			
1	4	4–5	4	4–5	4–5	4	4–5	3–4	4–5	4–5
2	4–5	4–5	4–5	3–4	3–4	4–5	4	4–5	3–4	4
3	4	4	4	3–4	3–4	4	4–5	4	4	3–4
4	4–5	4–5	4–5	4–5	4–5	4–5	4–5	4–5	4–5	4–5
5	4–5	4	4	4–5	4–5	4–5	4	4	4–5	4–5
6	4	4–5	4	3–4	3–4	4	4–5	3–4	4	3–4
7	4	4	4	4–5	4–5	4	4	4	4–5	4–5

Where, Alt = alteration St. = staining

**Fig. 6:**
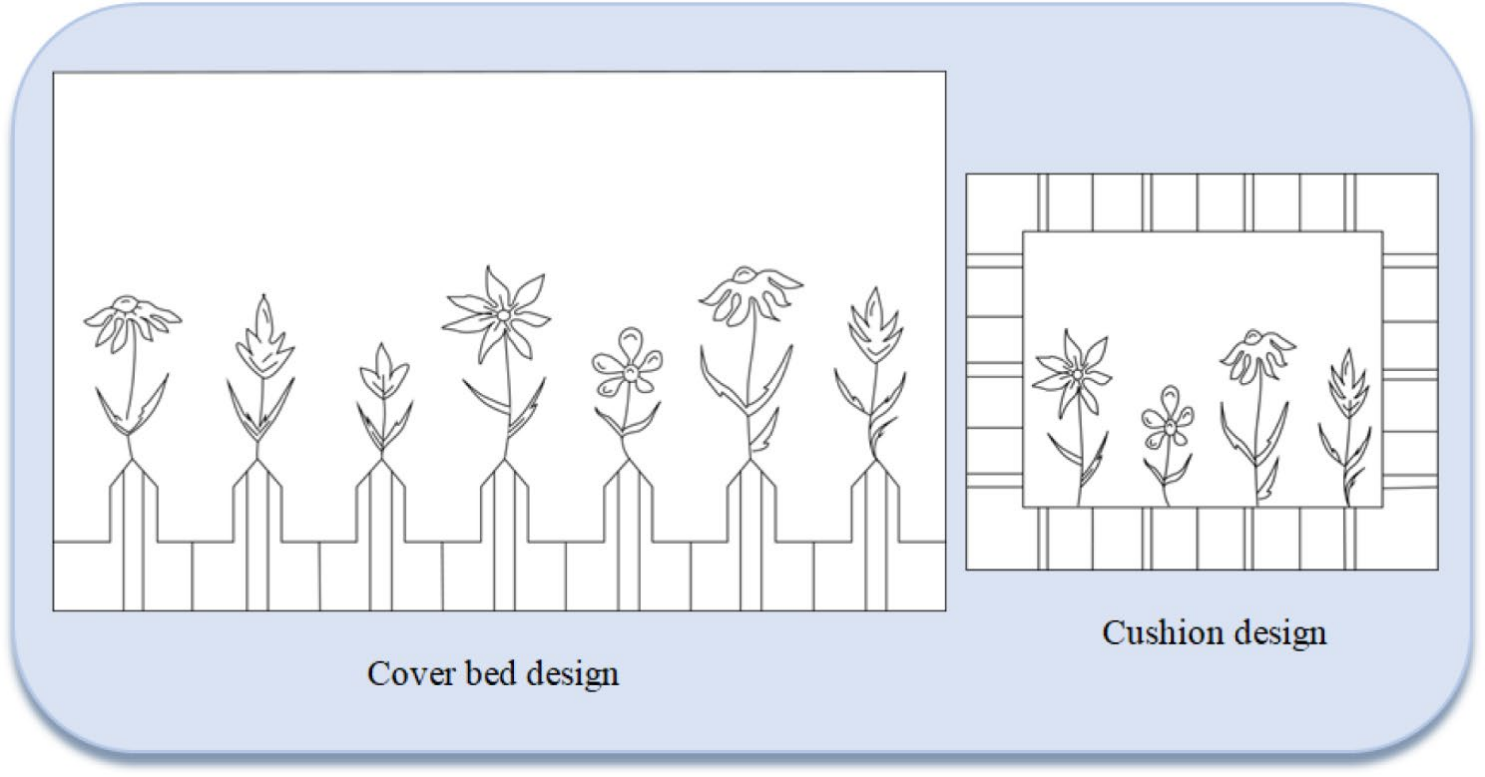
Applied design with Adobe Illustrator program

**Fig. 7:**
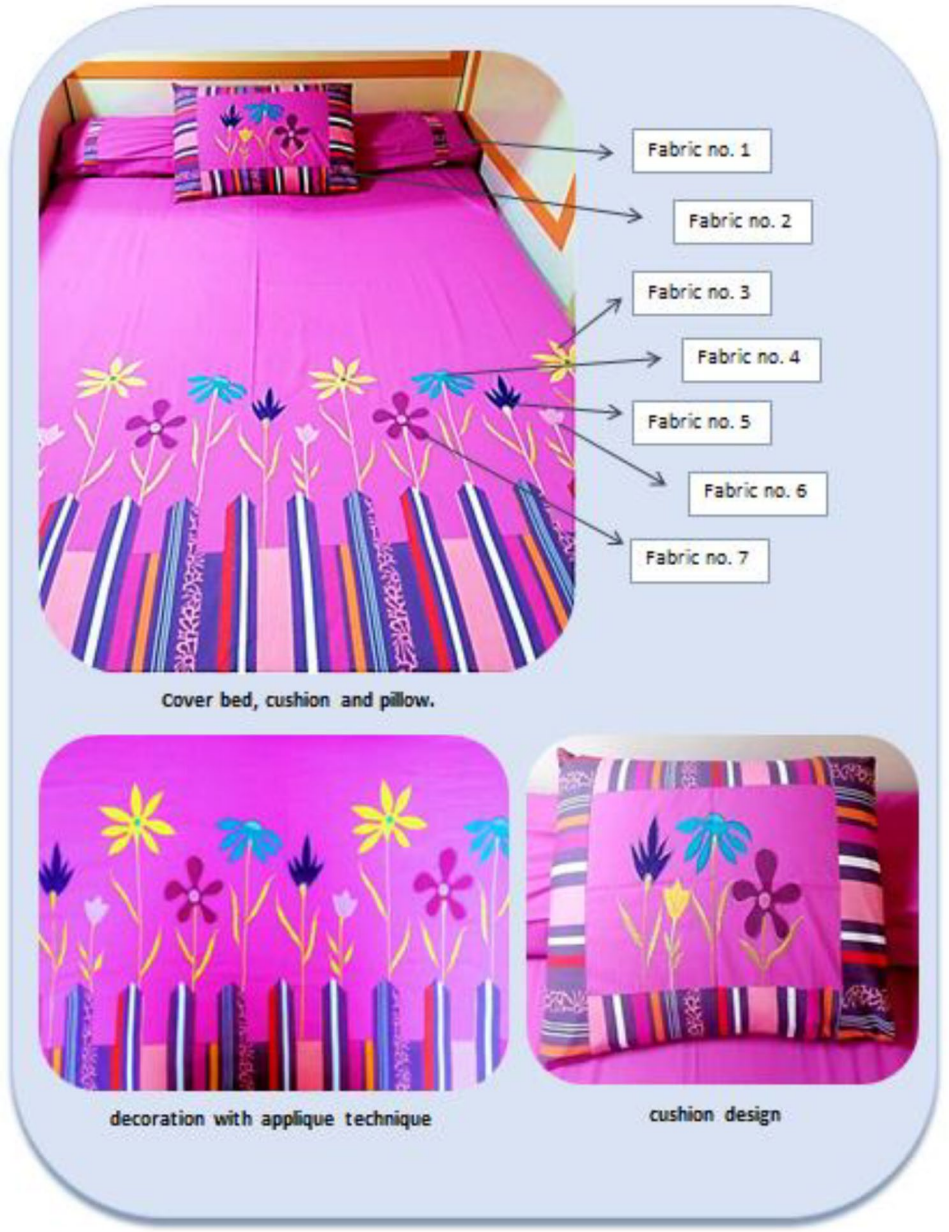
The applied design from collected matching waste by applique technique

Through previous results for all tests for collected waste fabric, the efficiency of these fabrics and their suitability for the design of cover beds and cushions was confirmed to apply with applique technique with half automatic embroidery method as shown in Figs. 6 and 7.

## Conclusion

Today recycling and reusing waste is not entertaining, but it become an important process to save the environment. Therefore, reusing waste is one of the major solutions, especially when applied to increase the national income. In this study, the waste fabric was subjected to treatment using nanocomposite-based chitosan and ZnONPs to function and produce value and high-quality home furnishings. Additionally, the prepared nanocomposite that based on chitosan and ZnONPs which was prepared in situ presented informative physiochemical and topographical results affirmed the green synthesis of ZnONPs and formulation of nanocomposite. The HR-TEM measured the ZnONPs at about 26 nm and DLS measurements affirmed the nanosize of nanocomposite particles with homogeneous and stable colloidal solutions. Moreover, the topographical analysis was amphizoid the attachment of nanocomposite over the fabric fibers with homogeneous distribution. This fabric was used for the formulation of home furnishing with multifunction properties such as excellent mechanical and antimicrobial activity properties after ten cycles of washing.

### Electronic supplementary material

Below is the link to the electronic supplementary material.

Supplementary Material 1

## Data Availability

The datasets generated during and/or analyzed during the current study are available from the corresponding author upon reasonable request.
