# Supplementary figures and images for: Design, characterizations, and antimicrobial activity of sustainable home furnishing-based waste fabric treated using biobased nanocomposite
Source: Bioresour Bioprocess. 2024 Jul 25;11(1):75. doi: 10.1186/s40643-024-00787-z (PMC11272763; doi:10.1186/s40643-024-00787-z)

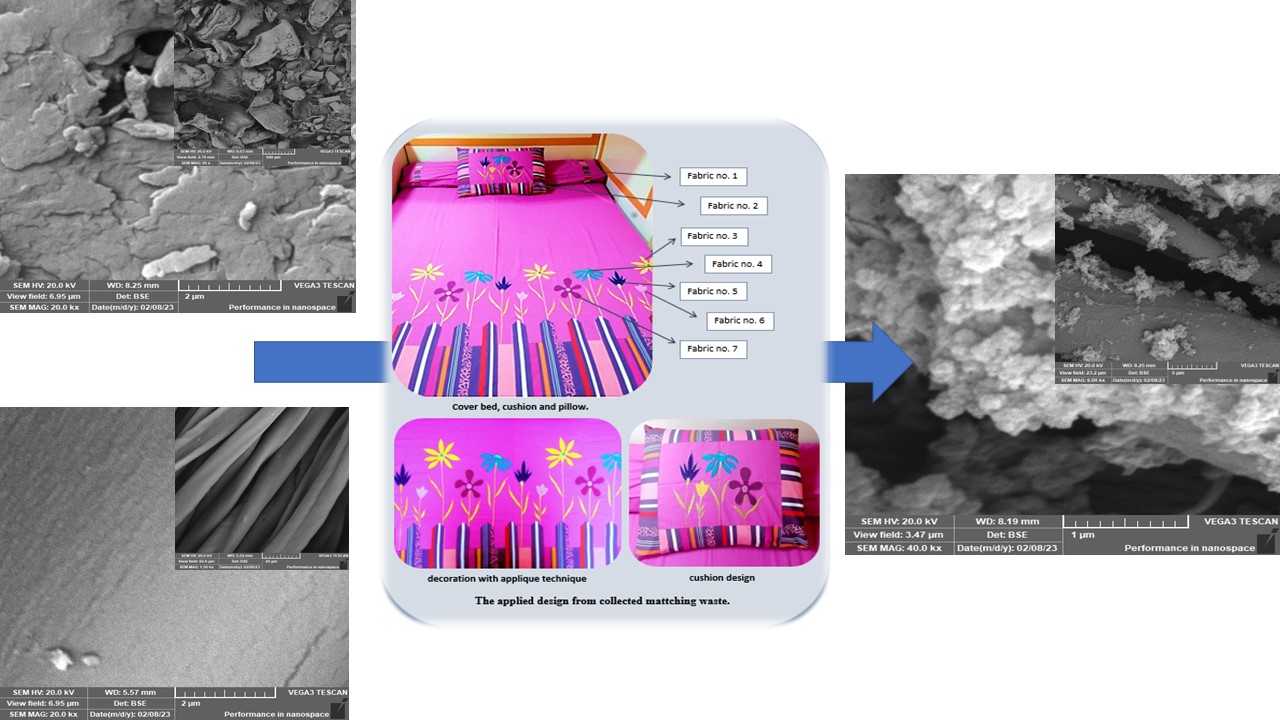

Supplement: Supplementary file 1 — Supplementary Material 1 [file 40643_2024_787_MOESM1_ESM.jpg]
